# Impact of Thermal Processing on the Structure, Antioxidant Properties and Hypoglycemic Activities of Sweet Potato Polysaccharides

**DOI:** 10.3390/foods13193082

**Published:** 2024-09-27

**Authors:** Chuan Liu, Yu Miao, Wenjia Zhou, Yiming Ma, Wenkui Guo, Aili Li

**Affiliations:** 1 Key Laboratory of Dairy Science, Ministry of Education, College of Food Science, Northeast Agricultural University, Harbin 150000, China; lc1397623595@163.com (C.L.); miaoyu04040303@163.com (Y.M.); s221002143@neau.edu.cn (W.Z.); swaggyaurora@126.com (Y.M.); 2 Heilongjiang Green Food Science Research Institute, Harbin 150000, China

**Keywords:** thermal treatment, sweet potato, polysaccharide, structure, antioxidant, hypoglycemic

## Abstract

In this study, three kinds of thermal treatments were applied to sweet potatoes: steaming (100 °C, 20 min), frying (150 °C, 10 min), and baking (200 °C, 30 min). We analyzed the changes in the physicochemical structure, antioxidant properties, and hypoglycemic activities of sweet potato polysaccharides between untreated and heat-treated samples. The results showed that the polysaccharides of all sweet potatoes (untreated and heat-treated) were composed of pyranose structures, had low protein content, and shared the same monosaccharide composition. Infrared spectra showed that the three thermal processing treatments had no significant effect on the functional groups or chemical bonding of sweet potato polysaccharides. In addition, all four polysaccharides exhibited dose-dependent antioxidant and hypoglycemic activities. The above experimental results suggest that thermal processing did not affect the physicochemical, antioxidant, or hypoglycemic activities of sweet potato polysaccharides.

## 1. Introduction

Thermal processing causes a series of changes in food ingredients. However, excessive heat treatment can destroy the nutrients in the food, such as proteins and polysaccharides [1] In recent years, people have been paying more attention to health, and the effect of heat treatment on food’s nutritional and functional characteristics has received extensive attention from research scholars.

Common heat treatment methods include boiling, steaming, high temperature and pressure, and deep-frying. Different thermal processing methods may have different effects on the nutritional and biological activities of food. Studies have shown that boiling dissolves water-soluble substances in food, such as sugars [2], resulting in a decrease in their content. Steaming has a lower effect on the water-soluble components of food than boiling [3]. Frying reduces the amount of soluble sugars, phenolics, and VC in foods [4]. For example, Cai et al. [5] conducted three types of thermal processing treatments, namely, steamed (100 °C, 5 min), fried (150 °C, 2 min), and baked (200 °C, 10 min), to comparatively analyze the changes in the content and structure of polysaccharides in scallops before and after thermal processing. The results showed that thermal processing treatments resulted in a significant increase in the base-soluble polysaccharide content of scallops, while steaming and frying treatments significantly decreased the water-soluble polysaccharide content (*p* < 0.05), and baking thermal processing resulted in a significant increase of 23.27% (*p* < 0.05). Heat processing treatments had no remarkable influence on the chemical bonding of scallop polysaccharides. The evidence suggests that the mode of heat processing has an important effect on both the physicochemical properties and the biological activity of the food.

Sweet potato belongs to the Convolvulaceae family [6]. Currently, sweet potato is the seventh most vital food worldwide, with the proportion of cultivation in Asia and Africa accounting for 95% of total global production [7]. China’s annual sweet potato output in 2014 was 56.017 million tons, while by 2020, sweet potato production fell to 51.264 million tons, of which the proportion of fresh sweet potato processing is about 50%, and the processing output of sweet potato starch-related products is about 1.5 million tons. Sweet potatoes are receiving a lot of attention from the food industry, consumers, and scientists due to the presence of various bioactive substances as an ingredient in health products and functional foods [8]. These unique chemicals provide multiple health benefits that promote health and longevity among consumers [9].

Plant polysaccharides have been found to possess a variety of biological activities, such as immunomodulatory, antioxidant, antimicrobial, anticancer, and anti-inflammatory activities [10,11]. Currently, research on sweet potatoes focuses on their stems and leaves, and recent studies have included their major bioactive components (such as polysaccharides) and their possible health benefits [12]. Sweet potatoes have been the major food source of carbohydrates for centuries, and today, they are also recognized as an effective food for the prevention and relief of many diseases. However, according to the data, few studies have been reported at this stage on the effects of thermal treatment methods on the structural characterization, physicochemical characteristics, and antioxidant and hypoglycemic activities of sweet potato polysaccharides. Therefore, three common thermal processing methods, namely steamed, fried, and baked, were used in this study to treat sweet potatoes. First, crude polysaccharides were isolated from sweet potatoes for structural characterization and physicochemical analysis. Then, the effects of different heat treatments on the in vitro antioxidant and hypoglycemic activities of the polysaccharides were investigated. This study provides a reference basis for rational thermal processing and functional product development of sweet potatoes. This research aimed to investigate the specific effects of thermal treatments on the structure, antioxidant, and hypoglycemic activity of sweet potato polysaccharides.

## 2. Materials and Methods

### 2.1. Materials and Chemicals

The experiment selected 20 kg of ripe sweet potatoes from Heilongjiang (Heilongjiang, China).

The following reagents were used in the research: Galacturonic acid, Beijing Biotope Technology Co., Shanghai, China; 1,1-Diphenyl-2-pyridylhydrazine (DPPH), Sigma, Cream Ridge, NJ, USA; Monosaccharide standard samples, CNW/IsoReag/TCI Inc; Ascorbic acid, Sinopharm Chemical Reagent Co., Shanghai, China; Acarbose, Shanghai Macklin Biochemical Technology Co., Shanghai, China; α-Glucosidase, Shanghai Aladdin Biochemical Technology Co., Shanghai, China; *p*-Nitrophenyl-α- d-glucopyranoside (PNPG), Shanghai Yuanye Biotechnology Co., Shanghai, China; α-Amylase, DNS reagent, BCA kit, Beijing Solebaum Technology Co., Beijing, China.

### 2.2. Preparation of Sweet Potato Powder

Before the preparation of sweet potato polysaccharides, the sweet potatoes need to be cut into pieces. This research group’s previous research can be referred to for the selection process of different heat treatment temperatures and times. The sweet potato powder was prepared using the method outlined in our previous study [1].

### 2.3. Extraction of Sweet Potato Crude Polysaccharide

The specific steps of sweet potato polysaccharide extraction can be referred to in the previous research of this research group [1]. The main process is as follows: First, the sweet potato powder is treated with ultrasound and stirred with α-amylase. After centrifugation, the supernatant is concentrated and precipitated with 95% ethanol (*v*/*v*) to obtain crude polysaccharide. After the polysaccharide was washed, the sweet potato crude polysaccharide was obtained by freeze-drying.

### 2.4. Determination of Monosaccharide Composition

#### 2.4.1. Sample Preparation and Extraction

Sample pretreatment: 20 mg of lyophilized sample was taken for the determination of polysaccharide fractions. In total, 500 µL of methanol, isopropanol, and water extraction solution were added to the sample. Then, the mixture was mixed and sonicated in a water bath at 4 °C for 30 min. It was centrifuged at 4 °C and 12,000 r/min for 3 min. Next, 20 µL (1000 µg/mL) of internal standard solution was added to 50 µL of supernatant and freeze-dried. The derivatization solution was obtained by incubating 100 µL of ammonium methoxypyridine at 37 °C for 2 h, followed by adding 100 µL of BSTFA and incubating at 37 °C for 30 min. *n*-hexane was added to 50 µL of the derived solution, diluted to 1 mL, filtered, and stored in a brown vial for GC-MS analysis [13,14].

#### 2.4.2. GC-MS Analysis

An Agilent 8890 GC (Agilent Technologies Co., LTD, Beijing, China) was used to detect the composition of acetylated monosaccharides. The column was a DB-5MS capillary column (30 m × 0.25 mm × 0.25 µm) with helium as the carrier gas. The temperature program was set according to Sun et al. [15].

### 2.5. Total Sugar, Total Protein, and Total Glucuronide Content

The phenol-sulfuric acid method was used for the measurement of total sugar content [16]. Protein content was determined using the BCA kit method [17]. Total glucuronide content was measured by the sulfate-carbazole method.

### 2.6. Fourier Transformation Infrared (FT-IR) Spectroscopy Analysis

To 2 mg of freeze-dried polysaccharide samples, dry KBr was added in a ratio of 1:100. After thorough grinding, the samples were pressed into thin slices using an electric tablet press. FT-IR (IS50, Bruker Instruments Co., Billerica, MA, USA) was performed in the wavelength range of 4000~400 cm^−1^.

### 2.7. Scanning Electron Microscopy (SEM) Analysis

The lyophilized polysaccharide sample fractions were ground into powder, an appropriate amount of the powder was taken, mounted on a copper sample holder, and sprayed with gold to treat the surface. The apparent morphology of the polysaccharide fractions was then observed using a scanning electron microscope (S3400N, Hitachi High-Technologies Co., Tokyo, Japan).

### 2.8. Determination of Zeta Potential and Particle Size

We adopted the method of Chen et al. [18] to determine zeta potential values and particle sizes of different sweet potatoes (2.0 mg/mL). These were measured using a Malvern Nano ZS90 Nanoparticle Size and Zeta Potential Analyzer (Malvern Panalytical Co., Shanghai, China) at room temperature.

### 2.9. Antioxidant Activity Assay

#### 2.9.1. DPPH Scavenging Assay

DPPH scavenging activity was measured using the method of Cumby et al. [19]. A volume of 2 mL 0.1 mmol/L DPPH was added to the polysaccharide sample. After mixing, the samples were protected from light at room temperature for 30 min, and the absorbance was recorded at 517 nm. The positive control was ascorbic acid (vitamin C, VC). The analysis was repeated three times for all samples and averaged. The formula is calculated as follows Equation (1)

(1) $$\mathrm{Scavenging}\;\mathrm{activity}\left(\%\right)=\left(1-\frac{A_{sample}-A_{blank}}{A_{control}}\right)\times 100\%\;$$

where $A_{sample}$ indicates 2 mL of sample and 2 mL of DPPH; $A_{blank}$ is the polysaccharide sample and 2 mL 95% ethanol; $A_{control}$ is the 2 mL 95% ethanol and 2 mL of DPPH.

#### 2.9.2. Hydroxyl Radical Scavenging Assay

Hydroxyl radical scavenging activity was adopted per Xu et al., [20] with slight modifications. In a test tube, 1 mL of polysaccharide solution, the same volume and concentration of ferrous sulfate solution, hydrogen peroxide solution, and salicylic acid solution were added sequentially (2 mL, 6 mmol/L), then incubated for 1 h in a 37 °C water bath. The absorbance was measured at 510 nm. The positive control was VC. The calculation formula is as follows:

(2) $$\mathrm{Scavenging}\;\mathrm{activity}\left(\%\right)=\left[\frac{A_{1}-(A_{2}-A_{3})}{A_{1}}\right]\times 100\%\;$$

where $A_{2}$ indicates the addition of sample solution and sodium salicylate solution; $A_{3}$ indicates the replacement of salicylic acid with deionized water; $A_{1}$ indicates that deionized water was used in place of the sample solution.

#### 2.9.3. Reducing Power Assay

The reducing capability of sweet potato polysaccharides was measured using the method reported by Zhu et al. [21]. A volume of 1 mL of the polysaccharide sample was added to a mixture of phosphate buffer (2.5 mL, 0.2 mol/L) and potassium ferricyanide (10 mg/mL) in a 1:1 (*v*/*v*) mixture. The mixture was incubated at 50 °C for 20 min, and then 2.5 mL trichloroacetic acid (100 mg/mL) was added. The mixture was centrifuged at 3000 r/min for 10 min. A volume of 2.5 mL of separated solution was mixed 1:1 (*v*/*v*) with distilled water, and 0.5 mL of ferric chloride (0.1%, *w*/*v*) was added. The absorbance was measured at 700 nm after 10 min. The analysis of all samples was repeated three times and averaged.

### 2.10. In Vitro Hypoglycemic Activity Assay

#### 2.10.1. Measurement of α-Amylase Inhibitory Activity

The method of Chen et al., [22] was adopted and modified for the determination of α-amylase inhibitory activity. A volume of 1.0 mL of polysaccharide solution, α-amylase solution (0.6 mg/mL, phosphate buffer pH 6.5), and starch solution (2.0 mg/mL) were mixed in a 1:1:1 ratio. The mixture was incubated at 37 °C for 20 min, and 5.0 mL DNS reagent was added. The mixture was then incubated in a boiling water bath for 5 min, and the absorbance was determined at 540 nm. Acarbose was used as the control group. The calculation formula is as follows:

(3) $$\mathrm{Inhibition}\;\mathrm{rate}\left(\%\right)=\left[\frac{A_{2}-(A_{x}-A_{3})}{A_{2}-A_{1}}\right]\times 100\%$$

where $A_{2}$, $A_{1}$, $A_{x}$ and $A_{3}$ are respectively the absorbance when the samples containing enzymes without polysaccharides, the samples containing enzymes without polysaccharides, samples containing enzymes and the blank (samples without enzymes).

#### 2.10.2. Measurement of α-Glucosidase Inhibitory Activity

The method of Li et al. [23] was used for the determination in this experiment. A volume of 40 μL of polysaccharide solution and α-glucosidase solution (0.04 U/mL) were mixed 1:1 and preheated at 37 °C for 5 min. Then, 20 μL PNPG (0.5 mmol/L) was added to start the reaction, incubated at 37 °C for 30 min, and then added 50 μL Na_2_CO_3_ solution (0.2 mol/L) to stop the reaction. The absorbance was measured at 405 nm. The formula is calculated as follows Equation (4).

(4) $$\mathrm{Inhibition}\;\mathrm{rate}\left(\%\right)=\left[1-\frac{A_{1}-A_{0}}{A_{2}}\right]\times 100\%$$

where $A_{0}$ contains polysaccharides but no α-glucosidase, $A_{1}$ contains polysaccharides and α-glucosidase, and $A_{2}$ contains α-glucosidase but no polysaccharides.

### 2.11. Statistical Analysis

All experiments were performed in triplicate. Experimental data were expressed as mean ± SD. SPSS was used for data processing and significance analysis. Originpro 2021 was used to produce the figures.

## 3. Results and Discussion

### 3.1. Polysaccharide Extraction

**Figure 1:**
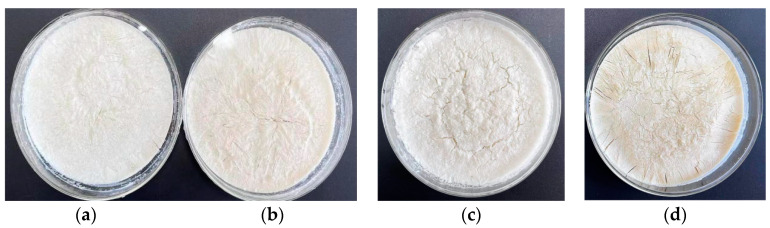
Plots of lyophilized samples of fresh sweet potato and three thermal processing treatments of sweet potato polysaccharides. (**a**) Fresh; (**b**) Steamed; (**c**) Fried; (**d**) Baked.

Four crude sweet potato polysaccharides were prepared from fresh, steamed, baked, and fried sweet potato samples, respectively, by the ultrasonic enzyme-assisted method. Figure 1 shows the morphology and color of lyophilized samples of sweet potato polysaccharides. As can be seen from Figure 1, the state of the steamed and fresh samples was similar, showing a relatively fine and loose powder. A morphological similarity was also observed between the fried and baked sweet potato polysaccharides, both showing the formation of hard sheets. The color of the baked sweet potato polysaccharide lyophilized sample was yellowish and darker than the other three samples. It was hypothesized that this may be caused by the Maillard reaction of the polysaccharide during heating. The yields of the four crude polysaccharides were 11.03% (fresh), 8.36% (steamed), 5.27% (fired), and 13.13% (baked). Wu et al., [24] found that the yield of purple potato polysaccharides extracted by the ultrasonic enzyme-assisted method was 5.42%. The yield of polysaccharides extracted from onion by Zhou et al. [25] was 4.96%. The reason for the variation in yield in this study may be the difference in variety, extraction temperature, extraction time, and solid-liquid ratio.

### 3.2. Physicochemical Properties of Polysaccharides

#### 3.2.1. Measurement of Monosaccharide Composition

**Figure 2:**
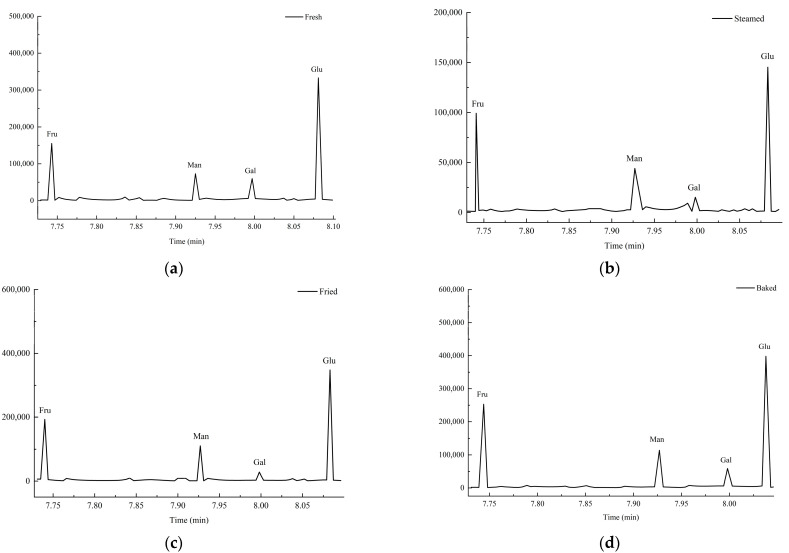
The monosaccharide composition spectrum of four polysaccharide samples. (**a**) Fresh; (**b**) Steamed; (**c**) Fried; (**d**) Baked. Note: Fru, D-Fructose; Man, D-Mannose; Gal, D-Galacturonic; Glu, Glucose.

Acetylated derivatives of fresh, steamed, fried, and baked sweet potato polysaccharides were identified by GC analysis (Figure 2). These four polysaccharides were found to consist primarily of fructose, glucose, mannose, and galactose.

The composition of monosaccharides is shown in Table 1. The results of the monosaccharide composition in this study are different from those in other studies. For example, Li et al. [26] described the monosaccharide composition of purple sweet potato polysaccharides by the hot water extraction method as containing rhamnose, arabinose, galactose, glucose, and glucuronic acid in a molar ratio of 1.89:8.45:1.95:1.13:1.0. It is speculated that the difference may be that the polysaccharides extracted in this study are mixtures and have not been purified.

#### 3.2.2. Determination of Total Sugar, Total Protein, and Total Glucuronic Acid Content

**Figure 3:**
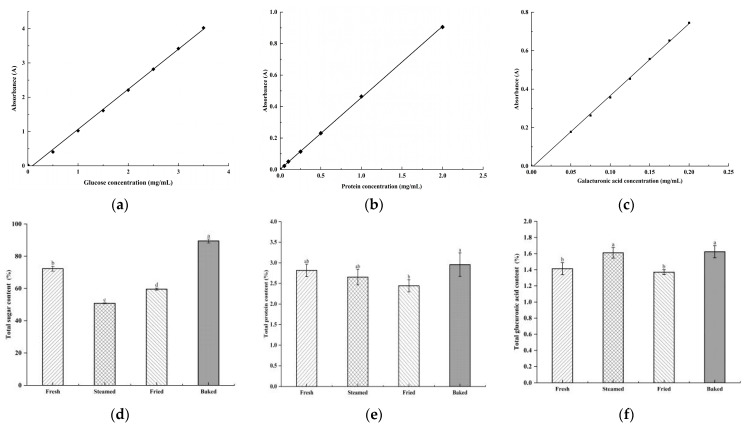
(**a**) the total sugar content measured for different thermally treated polysaccharides; (**b**) the protein content of the four sweet potato polysaccharides; (**c**) the total uronic acid content in the polysaccharides of steamed and baked sweet potatoes was remarkably different from that of fresh and fried sweet potatoes; Total sugar content (%) (**d**), total protein content (%) (**e**), and total glucuronic acid content (%) (**f**) of the four sweet potato polysaccharides. Note: Different lowercase letters indicate significant differences (*p* < 0.05).

Figure 3a shows the total sugar content measured for different thermally treated polysaccharides. It can be seen that the thermal processing treatments caused significant changes in the polysaccharide content of sweet potatoes (*p* < 0.05). The sweet potato polysaccharide content was significantly reduced by 22.24% and 44.44% after thermal processing by steaming and frying compared to the fresh group (*p* < 0.05). In contrast, there was a remarkable increase of 21.33% in polysaccharide content after baked thermal processing compared to the fresh group. Figure 3b shows the protein content of the four sweet potato polysaccharides. It was found that fresh sweet potato polysaccharides and the three thermal processing-treated polysaccharides contained some proteins, which were also found in other polysaccharides reported in the literature. For example, polysaccharides extracted from *Artemisia annua* [27] and purple sweet potatoes [28] also contained very low levels of proteins. Figure 3c shows that the total uronic acid content in the polysaccharides of steamed and baked sweet potatoes was remarkably different from that of fresh and fried sweet potatoes (*p* < 0.05). However, the differences between steamed and baked sweet potato polysaccharides, as well as between fresh and fried sweet potato polysaccharides, were not significant.

#### 3.2.3. Analysis of the Fourier Transform-Infrared Spectroscopy (FT-IR)

**Figure 4:**
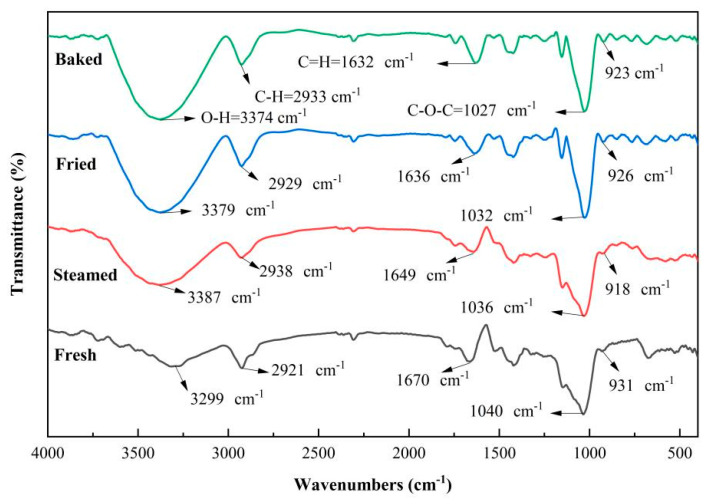
Infrared spectra of fresh sweet potato polysaccharides and three thermal processing treatments of sweet potato polysaccharides.

A more effective means of analyzing the structure of polysaccharides is infrared spectroscopy. Characteristic absorption peaks can show possible chemical bonds and functional groups [29]. The FT-IR spectra of the four sweet potato polysaccharides are shown in Figure 4. The intense absorption peaks at 3374, 3379, 3387, and 3299 cm^−1^ are attributed to the -OH stretching vibration, indicating the presence of hydroxyl groups in the four sweet potato polysaccharides [23,30]. The absorption peaks at 2933, 2929, 2938, and 2921 cm^−1^ are attributed to C-H stretching vibrations [23]. The C = O stretching vibration modes appeared at 1632, 1636, 1649, and 1670 cm^−1^ [31], and the glycosidic bond C-O-C stretching vibration modes were found at 1027, 1032, 1036, and 1040 cm^−1^ [30]. In addition, the absorption peaks between 1200 and 800 cm^−1^ for the four polysaccharides are characteristic of the pyran ring [30,31]. The region included peaks at 923, 926, 918, and 933 cm^−1^ due to asymmetric stretching of the pyran ring. The results showed that sweet potato polysaccharides are a type of pyranose, which is consistent with the results of purple sweet potato polysaccharides studied by [24].

The results showed no significant changes in the infrared spectra of sweet potato polysaccharides after different thermal processing treatments, indicating that heat processing has no remarkable influence on the characteristic functional groups and chemical bonds of sweet potato polysaccharides. Likewise, several other studies have reached a similar conclusion. For example, Li et al. [32] found that the infrared spectra of Pleurotus eryngii showed no significant changes in polysaccharide functional groups and chemical bonding after cooking treatment. Dong et al. [33] reported no significant difference in the infrared spectra of oat dextran before and after heat processing treatments and that the steaming and microwave treatments did not change the functional groups.

#### 3.2.4. Scanning Electron Microscopy (SEM)

**Figure 5:**
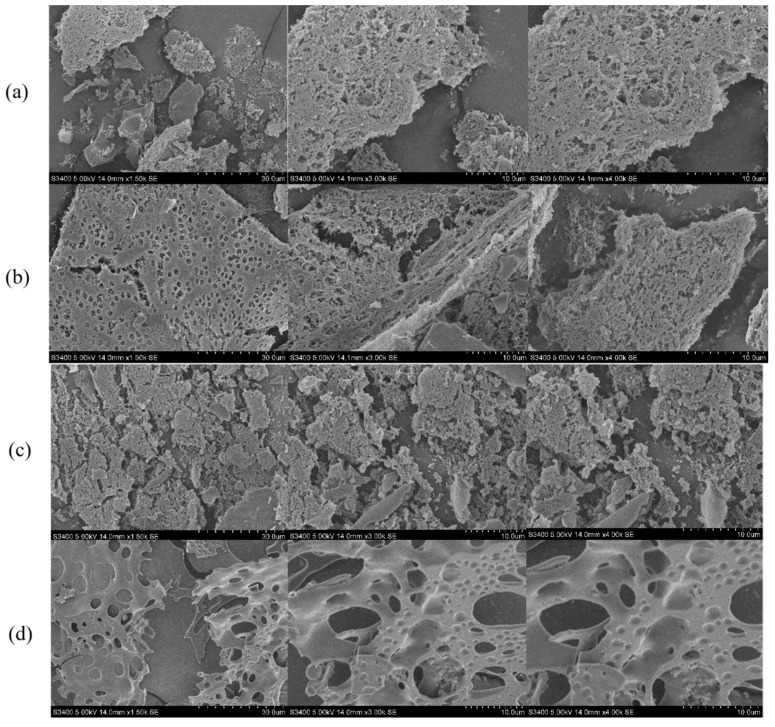
Scanning electron micrographs of raw sweet potato polysaccharides and three thermal processing treatments of sweet potato polysaccharides. ×1500, ×3000, and 4000. (**a**) Fresh; (**b**) Steamed; (**c**) Baked; (**d**) Fried.

The SEM images in Figure 5 show the differences in shape and color of the four polysaccharide samples. Consistent with the observations mentioned in Section 3.1, the morphology of the fresh sweet potato polysaccharides is similar to that of the steamed polysaccharide samples, with a distinctly flocculent surface, the presence of dense small pores, and a large lamellar structure. The surface of sweet potato polysaccharides after baking treatment was rough, with a broken mesh structure and uneven surface. This result is similar to that of the water-soluble polysaccharide of *Chlamys farreri* after baking, which showed a broken network structure and uneven surface [5]. In contrast, the surface of the fried sweet potato polysaccharide was smooth, showing irregular-sized bubbles as well as a large pore structure shaped like the surface of cheese, which is similar to that of enzymatically extracted *Salvia miltiorrhiza* polysaccharide [34]. The above evidence suggests that thermal processing treatments may disrupt the polysaccharide structure and rupture the cell wall, leading to denaturation and loss of nutrients. In addition, we also found that sweet potato polysaccharides formed a layer-on-layer structure. It can be seen from Figure 5 that fresh and heat-treated polysaccharides exhibit different SEM images.

#### 3.2.5. Measurements of Zeta Potential Value and Particle Size

The zeta potential value and particle size of a polysaccharide give some indication of the stability of the polysaccharide, thus determining the mobility and solubility of the polysaccharide in solution. In general, a smaller particle size of polysaccharide molecules in combination with a larger absolute value of zeta potential indicates that the polysaccharide molecules are more easily dispersed in the dissolution system. In contrast, it indicates that the polysaccharide molecules are more likely to aggregate and are not easily soluble [18]. Table 2 shows the average particle size and zeta potential values of the four sweet potato polysaccharides. There was a remarkable difference between the particle sizes of the four polysaccharides (*p* < 0.05). Notably, the particle size of the sweet potato polysaccharide in the untreated group was smaller, indicating that the sweet potato polysaccharide in the untreated group had relatively better solution stability. The zeta potential of the steamed sweet potato polysaccharide was not significantly different compared with the untreated group. Additionally, there was no significant difference in zeta potential between fried sweet potato and baked sweet potato. Still, the particle size of baked sweet potato was smaller, indicating that the solution of baked sweet potato polysaccharide was relatively more stable.

Acid or base treatment also has a certain effect on the stability of a polysaccharide solution. Zhong et al. [35] extracted polysaccharides from the wampee using hot water, hot acid, and hot alkali methods (WPP-W, WPP-A, and WPP). The smaller the polymer dispersity index (PDI) value, the more uniform the polysaccharide distribution. It was found that the PDI values of WPP-W and WPP-A were smaller, suggesting that WPP-W and WPP-A were more evenly distributed than WPP-AL. Among the three samples, WPP-W had the smallest PDI value and the largest absolute value of the average zeta potential, indicating that the WPP-W solution system was more stable.

### 3.3. Antioxidant Activity Assay

**Figure 6:**
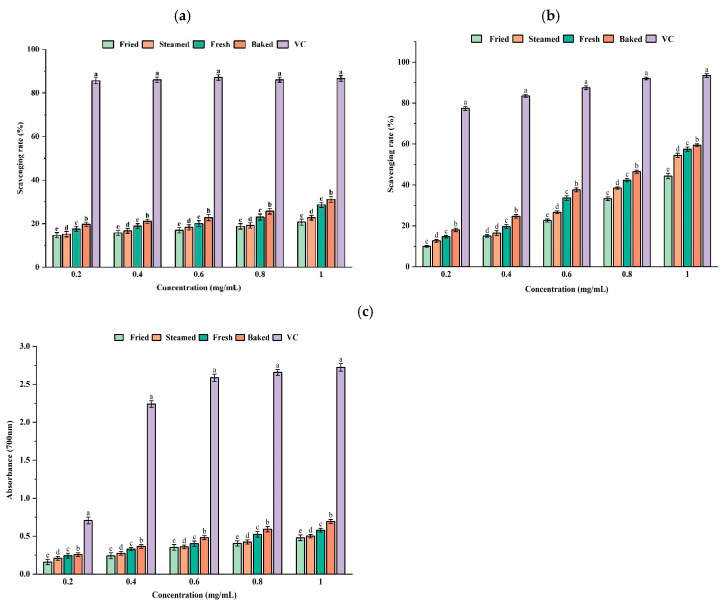
DPPH scavenging capacity (**a**), hydroxyl radical scavenging capacity (**b**), and reducing power (**c**) of four sweet potato polysaccharides. Note: Different lowercase letters indicate significant differences (*p* < 0.05).

Polysaccharides have antioxidant activity because of their free radical scavenging ability and reducing power [36]. Figure 6a shows untreated, and three heat-treated sweet potato polysaccharides exhibited a dose-dependent increase in DPPH radical scavenging ability. At the same concentration, the DPPH scavenging activities of the four sweet potato polysaccharides differed significantly (*p* < 0.05), with baked sweet potato polysaccharides having the strongest scavenging ability for DPPH radicals. These differences among the four polysaccharides may be related to their ability to donate hydrogen protons or electrons [37].

Hydroxyl radicals, as strong reactive oxygen radicals, can harm normal cells in the body and induce tissue damage. The antioxidant mechanism of polysaccharides may be that they act as hydrogen donors that bind to hydroxyl radicals to form stable radicals, thereby terminating free radical chain reactions. As shown in Figure 6b, all four sweet potato polysaccharides and ascorbic acid showed some scavenging ability for hydroxyl groups. At the same concentrations, the hydroxyl radical scavenging rate of the four sweet potato polysaccharides was significantly different, except at 0.4 mg/mL. (*p* < 0.05). The scavenging of hydroxyl radicals by fresh, steamed, fried, and baked sweet potato polysaccharides increased between the polysaccharide concentration range of 0 and 1.0 mg/mL. Among the four samples, baked sweet potato polysaccharides exhibited the most potent hydroxyl radical scavenging activity, which may be related to their ability to donate hydrogen protons.

Based on the reduction of oxidized intermediates, the reducing capacity can reflect the potential antioxidant activity of many components of food and chemicals in plants [38]. The reducing capacity of sweet potato polysaccharides was expressed as absorbance at 700 nm. Figure 6c shows the reducing power of the four sweet potato polysaccharides, showing their reducing power is concentration-dependent. Baked sweet potato polysaccharides had the strongest reducing power, while fried sweet potato polysaccharides had the weakest. At the same concentration, the reducing power of VC was significantly higher than that of the four sweet potato polysaccharides (*p* < 0.05). There were also significant differences in reducing power among the four sweet potato polysaccharides (*p* < 0.05). The above evidence suggests that sweet potato polysaccharides can be used as a potential natural antioxidant for use as a medicine or functional food. Yuan et al. [39] found that purple sweet potato polysaccharides significantly reduced oxidative damage, which was consistent with the results of the antioxidant activity of sweet potato polysaccharides in this study. Studies have reported that other polysaccharides also have a certain antioxidant capacity. As a recent example, Chen et al. [40] extracted crude polysaccharides from cushaw using the hot water extraction method and proved it had a good antioxidant effect.

### 3.4. In Vitro Hypoglycemic Activity Assay

**Figure 7:**
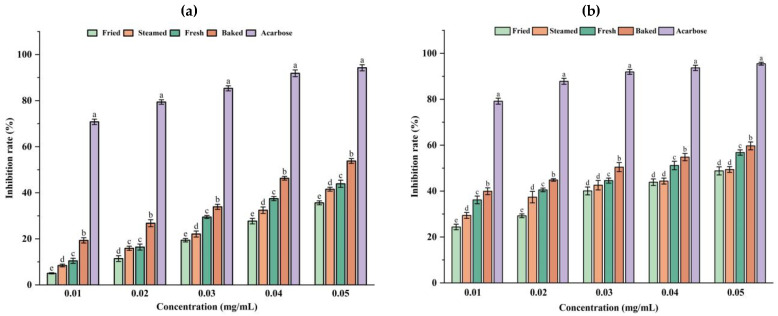
Inhibitory activities of four sweet potato polysaccharides on α-amylase (**a**) and α-glucosidase (**b**). Note: Different lowercase letters indicate significant differences (*p* < 0.05).

Alpha-amylase inhibitors and α-glucosidase inhibitors can delay the absorption of carbohydrates by reducing the amount of glucose in food. Alpha-amylase, a glycoside hydrolase, degrades starch into oligosaccharides or monosaccharides. The inhibitory effects of the four sweet potato polysaccharides on α-amylase are shown in Figure 7a. As the polysaccharide concentration increased, the inhibitory effect of the four polysaccharides on the activity of α-amylase increased gradually and showed a clear effect relationship. Furthermore, the inhibitory effects of baked sweet potato polysaccharides were greater than those of the fresh, steamed, and fried sweet potato groups (*p* < 0.05). However, all had a lower inhibitory effect than acarbose. The magnitude of the inhibitory effect of the four polysaccharides on α-amylase activity was baked > fresh > steamed > fried.

α-glucosidase exists in human intestinal mucosal cells and participates in human sugar metabolism, which can decompose carbohydrates into simple sugars easily absorbed by the human body, leading to a postprandial glucose increase in diabetic patients. However, alpha-glucosidase inhibitors can control postprandial blood sugar levels in diabetic patients by competitively inhibiting alpha-glucosidase activity and slowly breaking down carbohydrates into glucose. Figure 7b shows the effect of the four polysaccharides on α-glucosidase, all exhibiting a dose-dependent effect. At the same concentration, the inhibitory effect of baked sweet potato polysaccharides on α-glucosidase was significantly higher than that of fresh, steamed, and fried sweet potato polysaccharides (*p* < 0.05). The inhibitory effect of baked sweet potato polysaccharides on α-glucosidase at 0.05 mg/mL was 5.1% higher than that of the untreated group; however, it decreased by 15% and 16.4% in the steamed and fried groups, respectively. This indicated that the thermal treatment affected the hypoglycemic activity of sweet potato polysaccharides. Hu et al. [41] found that *Ginkgo biloba* seed polysaccharides extracted from *Ginkgo biloba* by different methods can significantly inhibit α-glucosidase activity. It is speculated that the reason may be that *Ginkgo biloba* seed polysaccharides reversibly or competitively bind to α-glucosidase in the small intestine brush border, slowing the decline of blood glucose levels. Another possible reason is that *Ginkgo biloba* seed polysaccharides have more surface charge and larger particles capable of binding to glucose, which further inhibits α-glucosidase activity.

In the clinic, acarbose is used as a type II diabetes mellitus treatment drug, which can effectively inhibit the activity of α-glucosidase. However, it can cause side effects such as bloating and diarrhea in patients. Therefore, although sweet potato polysaccharides do not inhibit α-glucosidase as much as acarbose, they are still expected to be developed as a safe and non-toxic potential inhibitor without side effects.

## 4. Conclusions

In this experiment, we investigated the effects of three thermal processing methods, namely steaming, baking, and frying, on the physicochemical properties, structural characterization, and antioxidant as well as hypoglycemic activities of sweet potato polysaccharides. The results of the research showed that steamed, baked, and fried treatments affected the physicochemical properties, antioxidant activity, and hypoglycemic activity of sweet potato polysaccharides. The polysaccharide content of steamed and fried thermally processed sweet potatoes was remarkably reduced compared to the fresh sweet potato samples. However, the polysaccharide content of baked sweet potatoes was significantly increased compared to the fresh group. The infrared spectra showed that the three heat processing treatments had no remarkable effect on the functional groups and chemical bonds of sweet potato polysaccharides. The antioxidant and hypoglycemic activities of sweet potato polysaccharides after thermal processing treatment were positively correlated with their total sugar content. The results of antioxidant and hypoglycemic activities showed that baked sweet potato polysaccharides had the highest antioxidant and hypoglycemic activity, while fried sweet potato had the lowest effect. This research provides a reference basis for the rational thermal processing of sweet potato and the development of functional sweet potato polysaccharide products.

## Figures and Tables

**Table 1:** The monosaccharide composition of four polysaccharides (molar ratios).

Monosaccharide Composition	Gal	Man	Fru	Glu
Fresh	1.0	1.6	52.1	82.3
Steamed	1.0	2.3	32.9	35.3
Fried	1.0	7.7	74.2	136.0
Baked	1.0	7.2	63.8	248.4

**Table 2:** Particle size and Zeta-values of sweet potato crude polysaccharide.

Particle Size and Zeta-Values	Fresh	Steamed	Baked	Fried
particle size (nm)	380.87 ± 3.59 ^d^	433.13 ± 14.52 ^c^	523.90 ± 11.63 ^b^	808.20 ± 32.34 ^a^
Zeta-values (mV)	−33.14 ± 1.11 ^b^	−34.42 ± 0.39 ^b^	−30.48 ± 0.65 ^a^	−30.29 ± 1.20 ^a^

Note: Different lowercase letters indicate significant differences (*p* < 0.05).

## Data Availability

The original contributions presented in the study are included in the article, further inquiries can be directed to the corresponding authors.
